# To Follow or Not to Follow: Social Norms and Civic Duty during a Pandemic

**DOI:** 10.1017/S0008423920000554

**Published:** 2020-06-08

**Authors:** Laura French Bourgeois, Allison Harell, Laura B. Stephenson

**Affiliations:** 1 Université du Québec à Montréal, 405 Rue Sainte-Catherine Est, Montréal, QC H2L 2C4; 2 University of Western Ontario, 1151 Richmond St, London, ON N6A 3K7

## Abstract

The outbreak of COVID-19 has put substantial pressure on individuals to adapt and change their behaviours. As the hope of a vaccine remains at least a year away, everyone is urged to take action to slow the spread of the virus. Thus, “flattening the curve” has become vital in preventing medical systems from being overrun, and it relies on massive collective action by citizens to follow specific public health measures such as physical distancing, hand washing, and physical isolation for vulnerable individuals. Despite the recommendations, the public has often been confronted with the reality that some individuals are not respecting them, including elected officials (Aguilar, 2020).

The outbreak of COVID-19 has put substantial pressure on individuals to adapt and change their behaviours. As the hope of a vaccine remains at least a year away, everyone is urged to take action to slow the spread of the virus. Thus, “flattening the curve” has become vital in preventing medical systems from being overrun, and it relies on massive collective action by citizens to follow specific public health measures such as physical distancing, hand washing, and physical isolation for vulnerable individuals. Despite the recommendations, the public has often been confronted with the reality that some individuals are not respecting them, including elected officials (Aguilar, 2020).

Media reports about individuals defying public health recommendations are not harmless (Jetten et al., 2020; Merkley and Loewen, 2020). Not only has recent framing ignored the very different circumstances facing parts of the population, but it also creates a sense of what “everyone else” is doing. Individuals make behavioural decisions by observing others, that is, by detecting social norms (see Steffens, 2020, for a discussion in the context of COVID-19). Reports of rule breaking can erode social norms of compliance: if it appears everyone else is breaking the rules, it becomes more acceptable to ignore public health recommendations oneself.

How to get a mass of people to participate in solving a collective problem when there are personal costs and few clear personal benefits has been a long-standing issue for scholars. From elections to public resource allocation, these situations require people to put collective concerns above their own. Research has shown that those who have a personal sense of civic duty are willing to make this trade-off. They believe that they have a moral obligation to act for the good of their community.

In this article, we explore the effects of civic duty and social norms on adherence to public health recommendations. We find that civic duty promotes following health recommendations and that norms become important in the absence of duty.

## Social Norms and Duty—Bringing Together Two Literatures

Exploring social norms and civic duty together in the context of the coronavirus pandemic is relevant because governments have launched massive campaigns—through discourse and policies—to encourage adherence to public health recommendations. Understanding what factors lead to adherence is central to success.

Social norms can be conceptualized as behaviours that characterize a sociocultural group (Sherif, 1936). They reflect how the majority of a group acts in particular instances. As social norms represent a consensus, they become rules for behaviour (Thibaut and Kelly, 1959) and useful guides to follow to receive acceptance from other group members (Prentice, 2012). Experimental and correlational studies have led to agreement—social norms lead to compliance (see Chung and Rimal, 2016, for a full review). The more people realize that a social norm exists, the more they are inclined to follow it (Biccheri, 2005).

Similarly, research on civic duty is compelling with respect to its impact on behaviour. Civic duty refers to a sense of responsibility toward society. Although it can affect a wide array of behaviours, it has mostly been studied in relation to voting (Blais, 2000). Researchers have demonstrated that individuals with a strong sense of civic duty are likely to vote because they perceive voting to be a moral obligation that they must abide by to be a good citizen (Blais and Daoust, 2020). However, there is limited research on the influence of civic duty in other contexts. For example, Blais, Galais and Mayer (2019) find that a duty to be politically informed is less prominent, and that for many who feel a duty to vote, their civic duty does not extend to being informed. In the current pandemic situation, little is known about whether civic duty includes conforming to behaviours such as physical isolation.

We focus on the roles of social norms and civic duty in compelling compliance with COVID-19 public health recommendations. We suspect each will encourage participation in following health guidelines, but that the effect of social norms will be strongest among those who do not see following the guidelines as a civic duty.

To evaluate these expectations, we make use of questions asked in the 2020 Democracy Checkup (Harell et al., 2020), gathered May 5–12, 2020.^1^ Norms were measured based on people's estimates of the percentage of Canadians who they “think are abiding by the public health recommendations aimed at slowing the spread of COVID-19” (0% to 100%). Civic duty was measured with a modified version of a question commonly asked about voting (Blais and Achen, 2019), where duty is coded 1 and choice 0:

– People have different views about the public health recommendations aimed at slowing the spread of COVID-19. For some, adhering to the recommendations is a duty, they feel they should follow them to help protect the health of Canadians. For others, adhering to them is a choice. For you personally, is adhering to the public health recommendations first and foremost a **duty** or a **choice**?^2^

Non-compliance was measured for four of the activities identified by the Government of Canada (2020) to stop the spread of COVID-19. Participants were asked about their behaviour in the previous week, including:

– Made non-essential trips in your community
– Gathered in groups with people outside your household
– Visited someone at higher risk, such as older adults and those in poor health
– Been closer than two arms lengths (about two metres) to someone outside your household

Response categories were no (0), only when necessary (1), and yes (2). “Only when necessary” was added to allow “face-saving” responses and gather more accurate measures (Daoust et al., 2020; Morin-Chassé et al., 2017). We combined the responses to the four questions into a single index for analysis (α = .63).

## Results

A large majority of participants (84%) believe that adhering to recommendations is a duty. A t-test analysis reveals that the participants who believe it is a duty to follow the guidelines are more likely to report that they did not engage in the four social activities measured (*M* = 0.39; *SD* = 0.42) compared to those who believe it is a choice (*M* = .56, *SD* = .51) (*t*(2002) = 6.43, *p* ≤ 0.001).

Turning to social norms, 77 per cent of the participants believe a majority of Canadians adhere to public health recommendations. Perceiving norm compliance does not predict whether individuals will comply themselves *r*(1967) = –.04, *p* = .09.^3^ Furthermore, only a weak relationship between perception of norm compliance and civic duty was observed (*r*(1919) = .05, *p* = .02), meaning that those who have civic duty are not necessarily inclined to perceive that other Canadians are similar to them and abide by public health recommendations. Because most people observe at least some compliance, we have recoded this variable into quartiles^4^ (0–60% = 1, 61–70% = 2, 71–80% = 3, and more than 80% = 4) for subsequent analysis.

**Figure 1:**
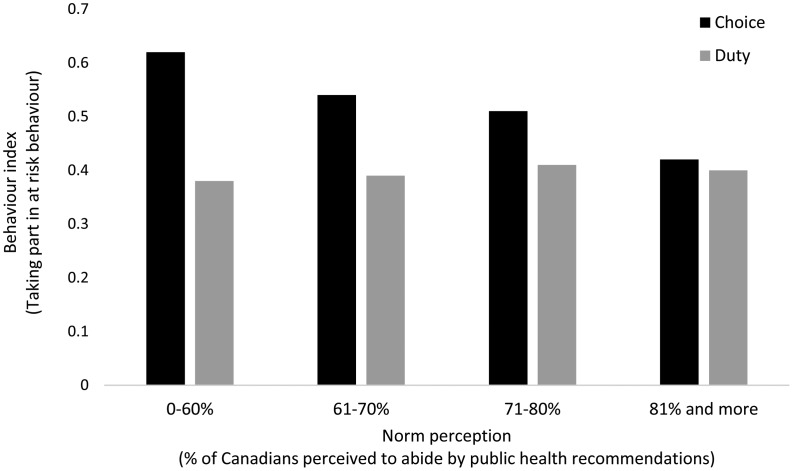
Norms and the moderating effect of civic duty.

To test whether compliance with public health recommendations varies depending on one's perceptions of civic duty and social norms, we used a moderation analysis conducted with 5,000 bootstraps in PROCESS (Hayes, 2017). We find that civic duty moderates the relationship between norms and rule-breaking behaviour (*F*(3, 1874) = 3.02, *p* =  0.03; see Table 1).^5^ We also observe direct effects for both duty and strong norms in Table 1, but as Figure 1 makes clear, norm perceptions do not matter to those with a sense of duty to follow health recommendations. However, those who see following the rules as a choice (about 16% of our sample) are less likely to report behaviour counter to current health recommendations if they perceive that 71 per cent or more of Canadians are following health guidelines.

**Table 1:** Model coefficients for moderation analysis

	Four-item behaviour index		
	Coef.	SE	*P*
Norm (ref = 60% or less)			
61–70%	–0.08	0.07	0.25
71–80%	–0.11	0.06	0.08
81% or more	–0.20	0.07	0.00
Duty	–0.24	0.05	0.00
Norm*Duty			
61–70%*Duty	0.09	0.08	0.24
71–80%*Duty	0.14	0.07	0.05
81% or more*Duty	0.22	0.07	0.00
Age in years	–0.01	0.00	0.00
University degree	–0.06	0.02	0.00
Woman	–0.08	0.02	0.00
COVID health worries	–0.04	0.01	0.00
COVID economic worries	0.00	0.01	0.85
Constant	1.17	0.07	0.00
N	1887		
R-Squared	0.08		

Note: Unstandardized coefficients are presented. Separate models are presented in Appendix Table A4 (available online) for each item in the behaviour index.

Looking at each behavior separately (Appendix, Table A4, available online), we find that civic duty has a strong direct effect on all four reported behaviours. Norms also have a direct effect on two of the four behaviors (gathering in groups and non-essential travel). As the moderation was significant for gathering in groups (*F*(3, 1865) = 6.80, *p* = 0.0001) and marginally significant for non-essential travel (*F*(3, 1857) = 2.37, *p* = 0.06), considering norms and civic duty together for these two behaviors explains more of the variation around rule-breaking than considering them separately.

## Conclusions

People's personal sense of duty is a strong predictor of pro-social behaviour in a crisis. When Canadians think following the rules helps society, civic mindedness fuels compliance and makes people almost immune to other people's (mis)behaviours. Yet, for those who see compliance with public health recommendations as a personal choice, perceptions of other people's behaviour become particularly important.

Our results speak to the importance of keeping government and media messaging focused on promoting rule compliance. A strong focus on rule breakers is likely to weaken compliance by eroding people's perceptions of other Canadians' behaviours. This study is only a snapshot of Canadians’ behaviours during a key moment in the COVID-19 crisis. Future research should explore who is most likely to perceive health recommendations as a duty to be followed and whether civic duty can be eroded by perceptions of norm noncompliance.

## Notes

1 See Appendix, Tables A1–A3 (available online), for further details. The 2020 Democracy Checkup is not yet publicly available; contact the authors for further information.

2 We recognize that this question does not provide a justification for “choice,” which may have led to overestimates of “duty.” This would make our findings conservative for those who feel duty and our findings somewhat generous for those who feel adherence is a choice. We do not think that this invalidates our conclusions given the strength of our results.

3 We looked for univariate and multivariate outliers as well as data normality. All were found to be within the acceptable limits.

4 Results for the full scale are available in Appendix, Table A5 (available online). The results are similar. Alternative cut points were also tested and made little substantive difference to our interpretation.

5 We controlled for education, worry about contracting COVID-19, worry about one's household economic situation, age, education and gender in the model.

## Supplementary material

For supplementary material accompanying this paper visit http://dx.doi.org/10.1017/S0008423920000554. click here to view supplementary material
