# Supplementary material for: To Follow or Not to Follow: Social Norms and Civic Duty during a Pandemic
Source: Can J Polit Sci. 2020 Jun 8:1–6. doi: 10.1017/S0008423920000554 (PMC7330279; doi:10.1017/S0008423920000554)
Supplement: Supplementary file 1 [file S0008423920000554sup001.docx]

Online Appendix

Data for this study was collected by the Consortium on Electoral Democracy (C-Dem) in their 2020 Democracy Check-Up Survey. The survey was fielded from March 5-12, 2020 with a sample of 8170 Canadian citizens and permanent residents, with independent quotas for age category, gender, and province, based on the 2016 Canadian Census, Catologue no. 98-400-X2016001. Data was collected on the Qualtrics platform, with a sample purchased from Dynata. The analysis reported in this article come from a random sample of 2071 panelists who were randomly assigned to receive questions about norms and attitudes related to the COVID-19 pandemic. We provide supplemental information about the analysis reported in the article here.

Table A1: Intercorrelations between variables

| Variables | 1 | 2 | 3 | 4 | 5 | 6 | 7 | 8 |
| --- | --- | --- | --- | --- | --- | --- | --- | --- |
| 1. Norm | 1.00 |  |  |  |  |  |  |  |
| 2. Duty | .05^*^ | 1.00 |  |  |  |  |  |  |
| 3. Behaviour index | -.04 | -.14^**^ | 1.00 |  |  |  |  |  |
| 4. Age in years | .17^**^ | .03 | -.19^**^ | 1.00 |  |  |  |  |
| 5. University degree | .01 | .07^**^ | -.05^*^ | -.08^**^ | 1.00 |  |  |  |
| 6. Woman | -.09^**^ | .02 | -.05^*^ | -.18^**^ | -.05^*^ | 1.00 |  |  |
| 7. COVID health worries | -.05^*^ | .15^**^ | -.11^**^ | -0.02 | 0.01 | .04^*^ | 1.00 |  |
| 8. COVID economic worries | -.12^**^ | -.01 | -.01 | -.06^*^ | -0.03 | .04^*^ | .31^**^ | 1.00 |
| Note. *N* = 2071. |  |  |  |  |  |  |  |  |
| ***p* < .01. |  |  |  |  |  |  |  |  |
| **p* < .05. |  |  |  |  |  |  |  |  |

Table A2 : Overview of Variables

| Variables | *M* | *SD* | *Min* | *Max* | *Obs* |
| --- | --- | --- | --- | --- | --- |
| 1. Norm (0-100) | 68.33 | 17.10 | 5.00 | 100.00 | 1974 |
| 2. Duty (0-1) | 0.84 | 0.37 | 0.00 | 1.00 | 2005 |
| 3. Behaviour index (0-2) | 0.42 | 0.44 | 0.00 | 2.00 | 2061 |
| 4. Age in years | 48.38 | 17.11 | 18.00 | 100.00 | 2071 |
| 5. University degree (0-1) | 0.52 | 0.50 | 0.00 | 1.00 | 2067 |
| 6. Woman (0-1) | 0.47 | 0.50 | 0.00 | 1.00 | 2064 |
| 7. COVID health worries (1-4) | 2.51 | 0.89 | 1.00 | 4.00 | 2056 |
| 8. COVID economic worries (1-4) | 2.37 | 0.93 | 1.00 | 4.00 | 2036 |

Table A3: Full question wording of control variables

| **Question** | **Answers** |
| --- | --- |
| *COVID health worries.* Thinking about the risk to you or your family of contracting COVID-19, would you say you are… | Not at all worried (0), A little worried (1), Somewhat worried (2), Very worried (4), Don’t know/ Prefer not to answer |
| *COVID economic worries*. How worried are you that your household's economic situation will be negatively affected by the coronavirus pandemic? | Not at all worried (1), A little worried (2), Somewhat worried (3), Very worried (2), Don’t know/ Prefer not to answer |
| *Education*. What is the highest level of education that you have completed? | No schooling, Some elementary school, Completed elementary school, Some secondary/ high school, Completed secondary/ high school,  Some technical, community college, CEGEP, College Classique, Completed technical, community college, CEGEP, College Classique  Some university, Bachelor's degree, Master's degree, Professional degree or doctorate, Don’t know/ Prefer not to answer  [Coded: No university degree (0), University degree (1)] |
| *Gender*. Are you... | A man (0), A woman (1), Other (eg. Trans, non-binary, two-spirit, gender-queer) [other was excluded from analysis] |
| *Age.* In what year were you born? | [Year] |

Table A4: Separate Models for Each Behaviour Item in 4-item Behaviour Index

|  | **Made Non-Essential Travel** | | |  | **Gathered in Groups** | | |  | **Closer than 2 metres** | | |  | | **Visited someone at risk** | | | |  |
| --- | --- | --- | --- | --- | --- | --- | --- | --- | --- | --- | --- | --- | --- | --- | --- | --- | --- | --- |
|  | Coef. | SE | *p* |  | Coef. | SE | *P* |  | Coef. | SE | *p* | |  | | Coef. | SE | *p* | |
| Norm (ref=60% or less) |  |  |  |  |  |  |  |  |  |  |  | |  | |  |  |  | |
| 61-70% | -0.06 | 0.11 | 0.61 |  | -0.27 | 0.09 | 0.00 |  | 0.00 | 0.12 | 0.97 | |  | | -0.02 | 0.08 | 0.80 | |
| 71-80% | -0.19 | 0.10 | 0.07 |  | -0.30 | 0.09 | 0.00 |  | -0.07 | 0.12 | 0.52 | |  | | 0.09 | 0.07 | 0.24 | |
| 81% or more | -0.30 | 0.11 | 0.00 |  | -0.34 | 0.09 | 0.00 |  | -0.18 | 0.12 | 0.13 | |  | | -0.02 | 0.08 | 0.84 | |
| Duty | -0.24 | 0.08 | 0.04 |  | -0.38 | 0.07 | 0.00 |  | -0.23 | 0.09 | 0.01 | |  | | -0.15 | 0.06 | 0.01 | |
| Norm*Duty |  |  |  |  |  |  |  |  |  |  |  | |  | |  |  |  | |
| 61-70%*Duty | 0.09 | 0.12 | 0.42 |  | 0.29 | 0.10 | 0.00 |  | -0.05 | 0.13 | 0.70 | |  | | 0.05 | 0.09 | 0.60 | |
| 71-80%*Duty | 0.23 | 0.12 | 0.05 |  | 0.35 | 0.10 | 0.00 |  | 0.07 | 0.13 | 0.59 | |  | | -0.05 | 0.08 | 0.55 | |
| 81% or more*Duty | 0.29 | 0.12 | 0.02 |  | 0.40 | 0.10 | 0.00 |  | 0.11 | 0.13 | 0.40 | |  | | 0.10 | 0.09 | 0.23 | |
| Age in years | -0.01 | 0.00 | 0.00 |  | -0.01 | 0.00 | 0.00 |  | -0.01 | 0.00 | 0.00 | |  | | -0.01 | 0.00 | 0.00 | |
| University degree | -0.11 | 0.03 | 0.00 |  | -0.06 | 0.03 | 0.02 |  | -0.04 | 0.03 | 0.20 | |  | | -0.04 | 0.02 | 0.09 | |
| Woman | -0.14 | 0.03 | 0.00 |  | -0.06 | 0.03 | 0.02 |  | -0.01 | 0.04 | 0.79 | |  | | -0.12 | 0.02 | 0.00 | |
| COVID health worries | -0.06 | 0.02 | 0.00 |  | -0.04 | 0.02 | 0.00 |  | -0.07 | 0.02 | 0.00 | |  | | -0.01 | 0.01 | 0.70 | |
| COVID economic worries | 0.02 | 0.02 | 0.27 |  | -0.01 | 0.01 | 0.41 |  | 0.00 | 0.02 | 0.92 | |  | | 0.00 | 0.01 | 0.85 | |
| Constant | 1.47 | 0.12 | 0.00 |  | 1.11 | 0.08 | 0.00 |  | 1.30 | 0.11 | 0.00 | |  | | 0.63 | 0.07 | 0.00 | |
|  |  |  |  |  |  |  |  |  |  |  |  | |  | |  |  |  | |
| N | 1870 | | |  | 1878 | | |  | 1876 | | |  | | 1881 | | | |  |
| R-Squared | 0.04 | | |  | 0.07 | | |  | 0.04 | | |  | | 0.05 | | | |  |

Table A5: Model for 4-item Behavior Index with the continuous norm variable

|  | 4 item index | | |
| --- | --- | --- | --- |
|  | Coef. | SE | *p* |
| Norm | -0.002 | (.001) | 0.11 |
| Duty | -0.29 | (.10) | 0.005 |
| Norm*Duty | 0.002 | (.001) | 0.10 |
| Age in years | -0.006 | (.0006) | 0.00 |
| University degree | -0.07 | (.02) | 0.001 |
| Woman | -0.08 | (.02) | 0.00 |
| COVID health worries | -0.05 | (.01) | 0.00 |
| COVID economic worries | 0.001 | (.01) | 0.90 |
| Constant | 1.14 | (.11) | 0.00 |
|  |  |  |  |
| N | 1887 | | |
| R-Squared | 0.08 | | |
